# Model Construction of Chinese Preservice Physical Education Teachers’ Perception of Social Media: A Grounded Theory Approach

**DOI:** 10.3390/ijerph20053886

**Published:** 2023-02-22

**Authors:** Yue Xu, Zhihua Yin, Haohui Liu, Mingzhu Sun, Zhen Guo, Bo Liu

**Affiliations:** 1 Department of Physical Education and Sport Sciences, University of Limerick, V94 T9PX Limerick, Ireland; 2 College of Physical Education and Health, East China Normal University, Shanghai 200241, China; 3 Department of Physical Education Teaching, Shanghai University of Engineering Science, Shanghai 201620, China; 4 Division of Sports Science and Physical Education, Tsinghua University, Beijing 100084, China

**Keywords:** preservice physical education teacher, social media, grounded theory

## Abstract

(1) Background: Pre-service physical education teachers commonly embrace social media for multiple purposes. However, little is known about their perception of social media, which could affect the appropriate use of social media in their future professional work. This study aims to explore a theoretical model of how pre-service physical education teachers perceive social media in order to provide a basis for educators to guide their appropriate use of social media. (2) Methods: Qualitative data were collected in diverse ways, mainly from interviews. Seventeen Chinese preservice physical education teachers were selected as participants by a purposive sampling technique. The interview questions focused on participants’ motivation, expectations, and experiences in social media usage. Grounded theory was used to analyze the data by ROST CM and Nvivo 12. (3) Results: The perception of social media among teachers includes three subsidiary categories made up of 10 sub-categories, 70 concepts, and 307 labels. The three categories are (a) value perception, including the perspective of intelligent function, interaction, and rich information, (b) risk perception, involving psychological risk, information risk and privacy risk and (c) overall perception, like development trends, current status and basic elements. (4) Conclusions: Chinese preservice physical education teachers perceive social media as having similarities and differences compared to other countries. Future research should consider a large sample survey to revise and verify the initial exploration of perception and study diverse groups of teachers’ perceptions of social media.

## 1. Introduction

Social media has emerged as a prevalent and widely used form of new media, embraced by individuals worldwide, with a special focus on the youth demographic. As for the youngest cohort of physical education teachers, preservice physical education teachers, who possess an insatiable curiosity but lack teaching guidance and experience, social media presents a double-edged sword of opportunities and challenges. On the one hand, social media offers various benefits, such as free access to professional knowledge, skills, and opinions, serving as a primary source for news retrieval, and providing avenues for global communication. As a result, social media has become an essential tool for professional development among physical education teachers [1]. On the other hand, the growing prevalence of fake news on social media is a concern, especially in fields such as physical education and health [2]. The dissemination of false information through social media can harm the reputation and credibility of preservice physical education teachers and have a negative impact on their professional standing. Therefore, it is crucial for preservice physical education teachers to be mindful of both the potential consequences of fake news and the benefits of utilizing social media in a responsible and informed manner.

The perception of social media among physical education teachers has received increasing attention in recent years, given its potential impact on their professional development. In order to gain a deeper understanding of social media in physical education, several studies have employed a grounded theory approach. This method is growing in popularity, especially in physical education. A few examples of studies that used grounded theory include Pummell, Harwood, and Lavallee (2008), who explored perceptions of within-career transitions among equestrian event riders [3], Goodyear et al. (2019), who studied the characteristics of a Twitter-based professional learning community called #pechat among physical education teachers [4], McNamara et al. (2021), who found that physical education teachers were more likely to engage in communication through social media for learning activities [5], and Hyndman and Harvey (2019, 2020), who examined preservice teachers’ perceptions of using Twitter in health and physical education teacher education [6,7]. The study by Goodyear et al. (2019) provides valuable insights into the potential of social media to support the professional development of physical education teachers, while McNamara et al. (2021) found that physical education teachers were more likely to engage in communication through learning activities on social media. Hyndman and Harvey (2019, 2020) determined that preservice teachers saw many values in using Twitter for health and physical education teacher education, such as autonomy, relatedness, and competence, but also raised concerns about undesired Twitter users and navigating the platform. As we can observe, the understanding of the perception of social media among physical education teachers is becoming increasingly comprehensive, however, the lack of representation from eastern countries makes the goal difficult to achieve.

Previous research has made a huge contribution to the field of social media and physical education teachers’ professional development, while the current investigation includes limitations related to the sample that should be acknowledged in interpreting the results [8]. The geographical location of previous research is unclear. Moreover, most of the research conducted and collected data is from the United States [1,5,7,8,9,10,11,12,13,14,15,16], followed by Australia [6,17], Spain [18,19,20], Ireland [21,22], and the United Kingdom [23,24,25,26], while only three related research included developing countries [27,28,29]. The research from eastern countries sees social media only as a data collection platform instead of in the context of teachers’ professional development [27] or has used questionnaire surveys to conduct the research [28]. A questionnaire survey is a statistical study to test a theory on social phenomena or human problems. It measures variables with numerical data and analyzes results to validate a theory [30,31]. To some extent, the questionnaire suits the research topic of teachers’ perceptions. Unfortunately, the perceptions among teachers are so complex and interwoven that they cannot be reduced to isolated variables and presented in detail. The perception of social media is complex, making it necessary to use a theoretical model to better understand it. Among various methods available, grounded theory is considered the best choice. Grounded theory allows researchers to create a theory based on participants’ experiences, perspectives, and behaviors, providing insight into how people perceive and make sense of social media. With its inductive data analysis, grounded theory enables researchers to develop a theory specific to the participants’ experiences and allows for an iterative process of data collection and analysis, leading to a deeper understanding of participants’ perceptions [32]. There is limited knowledge regarding the perception of preservice physical education teachers towards social media in China. If teachers lack a thorough understanding of social media, they may only use it for leisure or personal purposes rather than for their professional development. To address this, exploring a theoretical model of preservice physical education teachers’ social media perceptions could inform their understanding of social media and provide a scientific framework for effective professional development. Also, the potential for generalizability from an in-depth study was addressed. A wider sample could have provided further insights [4] and explored similarities and differences in different groups of teachers [9]. China is one of the biggest countries training preservice physical education teachers. Specifically, in mainland China, there are 1423 colleges and 1265 universities totaling 2688 higher education institutions, and 317 of these universities offer physical education teacher education (PETE) programs for preservice physical education teachers [33].

Chinese preservice physical education teachers are a large group of members of social media users for their professional development and use different social media platforms compared to teachers from western countries. However, little is known about Chinese preservice physical education teachers’ perceptions of their own social media usage. It is obvious that different culture differences exist among different countries, which suggests a need to explore the differences instead of totally copying the experience from other countries. Such information is theoretically significant given the paucity of knowledge about this professional group. It can also have practical implications for future planning for international collaborative professional development and identifying the specific challenges or professional needs among preservice physical education teachers. Given the scholarly attention afforded to social media to promote professional development among physical education teachers, it is necessary to fill the research gap, especially in terms of evidence, population, and methodology. To ameliorate these weaknesses, we used grounded theory, which is well-suited for perception research because it allows researchers to develop a theory that is grounded in the experiences and perspectives of the participants, provides an inductive approach to data analysis, and allows for an iterative process of data collection and analysis. We also focus on a Chinese sample, which has the largest population of physical education teachers.

In summary, the utilization of social media among preservice physical education teachers has increased, yet the comprehension of their perceptions regarding the usage of social media remains limited, particularly in China. The aim of this study is to address this gap by exploring preservice physical education teachers’ perceptions of social media and developing a theoretical model to capture these perceptions. This study seeks to contribute to the advancement of knowledge concerning social media in physical education teacher education, providing valuable insights for teacher educators and educational institutions.

## 2. Materials and Methods

As mentioned above, pre-service physical education teachers frequently use social media for various purposes. Nevertheless, their perceptions of social media are not well-understood, and this lack of understanding could impact their ability to use social media appropriately in their future professional endeavors. Thus, our research question is what is the perception of social media among Chinese preservice physical education teachers? Our research objective is to develop a theoretical model of social media perception among Chinese preservice physical education teachers. Specifically, grounded theory is an effective approach for developing the perception model of social media, as it is based on participants’ experiences, provides inductive data analysis, and allows for the refinement of understanding as data is collected. The basic logic of grounded theory is to condense data from the bottom up and establish a theory [34]. Researchers do not make assumptions beforehand but instead initiate data induction analysis directly. As a result, the relevance and validity of the data must be ensured during data collection.

This study collected data through online posts, literature, and interviews. First, a literature search was conducted using domestic and international databases such as CNKI (China National Knowledge Infrastructure) and Web of Science to gather information on the perception model of social media among pre-service physical education teachers. A total of 48 Chinese and 203 English relevant articles were obtained and analyzed to extract preliminary concepts and support the theoretical framework developed in the text. Additionally, online posts related to physical education, such as #pechat and #pe, were collected from popular social media platforms (WeChat, Twitter, TikTok, etc.). Finally, semi-structured interviews were conducted with 17 pre-service physical education teachers. The details of the interviews are described below.

### 2.1. Participants

It is important to note that the appropriate sample size in grounded theory research is determined by the data and not by a specific number of participants. The sample size should be sufficient to generate a rich and diverse data set that allows the researcher to develop a theory that is grounded in the data. The sample size was based on the criterion of theoretical saturation; that is, no new relevant data emerged regarding a category, categories were well-developed, and relationships among categories were established and validated [32]. In the field of physical education, studies demonstrate that a sample size of 10–20 participants can be sufficient in grounded theory research [35], particularly when the aim is to generate a rich and detailed understanding of a particular phenomenon. Hence, 17 participants were selected to be interviewed, which meets the requirements of grounded theory.

Following ethical approval, participants were recruited through a combination of purposeful and snowball sampling [36]. Participants were sourced from every class level and every type of university, and every part of China (East–North, East–South, West–North, West–South). Hence, it can be inferred that the participants have a plausible representation of the target population. The study employed three revised inclusion criteria from Goodyear (2019) [4] to select participants: (1) current active use of social media (defined as frequent daily use); (2) a minimum of five years of experience using social media; and (3) utilization of social media for multiple purposes, including professional development. In order to preserve the anonymity of participants, each was assigned a numerical identifier from 1 to 17 (refer to Table 1 for details).

### 2.2. Interview

Ethical approval was approved by the research ethics committee of the authors’ institute. A conversational, in-depth interview was guided by six interview questions (see Table 2). These interview questions were modified from a previous study [4,11,34]. The interview questions were initially developed based on the goals of the study and were discussed with three professors in the field of physical education teacher education (PETE). After several revisions, the final interview outline included six open-ended questions. These questions covered two main topics: (1) participants’ perceptions of the characteristics and uses of social media and (2) their perceptions of the challenges, benefits, and preferences related to using social media. Due to the COVID-19 pandemic, the 17 individual semi-structured interviews were conducted online. At the beginning of each interview, we followed previous studies and established a trusting relationship with the participants. This was done in order to ensure that the participants felt comfortable and willing to share their thoughts and experiences with us. By creating a positive and open environment, we were able to gather rich and valuable data that helped us better understand the topic at hand [37]. To put the participants at ease, we engaged them in casual conversation. This included discussing hot topics on microblogs, explaining funny emojis on social media, and highlighting the importance of social media usage. The interviews lasted an average of 60 min, and all participants agreed to have their interviews recorded. This allowed us to capture their responses accurately and in full, which was crucial for our analysis.

### 2.3. Data Analysis

When all interviews were completed, the recordings were transcribed verbatim by ROST CM 6 software [38], and NVivo 12 [39] was used to store and manage the data. The data were analyzed using constant comparative analysis, and the transcripts of each interview were carefully reviewed. Initial evaluation of the data was followed using grounded theory, including the steps of open coding, axial coding, and selective coding to analyze the qualitative data. This systematic approach allowed us to identify key themes and patterns in the data, which provided insights into participants’ perceptions of social media and its use in the context of physical education teacher education [40]. The data for this paper were collected from a variety of sources, including literature on social media perception among physical education teachers, social media posts related to physical education, and semi-structured interviews with preservice physical education teachers. These data were then analyzed using a coding process to identify key themes and patterns, which allowed us to form a comprehensive understanding of participants’ perceptions of social media in their professional fields. The resulting research provides insights into the potential challenges and benefits of using social media in Chinese physical education teacher education, as well as suggestions for future research on physical education teacher education [41].

#### 2.3.1. Open Coding

Open coding refers to the initial conceptualization, further conceptualization and categorization of raw empirical data [40] related to preservice physical education teachers’ social media perceptions. The open coding process was used to identify key concepts and categories related to preservice physical education teachers’ perceptions of social media. This involved capturing keywords that appeared in primary sources related to these perceptions and using native words from empirical sources to form free nodes for preservice physical education teachers’ social media perceptions. This allowed us to build a comprehensive understanding of the key themes and patterns in the data and to develop a theoretical framework for our research.

Step 1: Labeling and Conceptualization. Through the open coding process, we identified 307 labels related to preservice physical education teachers’ perceptions of social media. These labels were then subjected to constant comparative analysis, in which they were compared with each other to identify key themes and patterns. This process, guided by grounded theory, allowed us to condense and refine the labels, resulting in 70 exclusive concepts that captured the essence of preservice physical education teachers’ social media perceptions. These concepts provided a solid foundation for our theoretical framework and helped us to better understand the data we had collected before (see Table 3 and Table 4).

Step 2: Categorization. Using the same process of constant comparative analysis, we further classified and compared the 70 concepts we had identified. This allowed us to identify 10 subsidiary categories that captured the key themes and patterns in the data. These categories included Media Interface Perception, Privacy Risk Perception, Information Value Perception, Basic Element Perception, Current Status Perception, Information Risk Perception, Intelligent Function Perception, Development Trend Perception, Interactive Value Perception, and Psychological Risk Perception. These categories provided a more detailed and nuanced understanding of preservice physical education teachers’ perceptions of social media and helped us to develop a comprehensive theoretical framework for our research. (Seen in Table 5).

#### 2.3.2. Axial Coding

Axial coding refers to the categorization and comparison of various concepts or categories related to preservice physical education teachers’ perceptions of social media. This process involves breaking down and reorganizing the free nodes of these perceptions to extract the main concepts or categories. In axial coding, we dismantled the free nodes of preservice physical education teachers’ social media perceptions and reorganized them in a way that allowed us to analyze and conceptualize these perceptions in a more systematic and comprehensive manner. This helped us to identify key themes and patterns in the data and to develop a more robust and nuanced theoretical framework for our research.

Through further constant comparative analysis, we condensed the 10 subsidiary categories identified in the axial coding process into three main subcategories: Overall Perception, Value Perception, and Risk Perception. Each of these subcategories included different subsidiary categories from the original set. Specifically, the “Overall Perception” subcategory included “Interface Perception”, “Element Perception”, “Current Status Perception”, and “Development Trend Perception”; the “Value Perception” subcategory included “Information Value Perception”, “Interactive Value Perception”, and “Intelligent Function Perception”, and the “Risk Perception” subcategory included “Psychological Risk Perception”, “Privacy Risk Perception”, and “Information Risk Perception”. These subcategories provided a more refined and focused view of preservice physical education teachers’ perceptions of social media and helped us to develop a more comprehensive and nuanced theoretical framework for our research. (See Table 6 for details).

#### 2.3.3. Selective Coding

Selective coding is the process of analyzing the links between the different main concepts or categories [40] of pre-service PE teachers’ social media perceptions and making continuous comparisons to further uncover the ‘core categories’ that can unify all pre-service physical education teachers’ social media perception concepts or categories. Selective coding is the final stage of the grounded theory analysis process, in which the data are analyzed in relation to the key themes and patterns identified in the earlier stages of coding. This process involves making connections between the data and the concepts and categories that have been identified and using memos and other analytical tools to develop a comprehensive theoretical framework for the research. In the case of this study, selective coding helped us to understand the key factors that influence preservice physical education teachers’ perceptions of social media and to develop a theoretical model that captures the complex and nuanced nature of these perceptions.

Through the continued examination of the 10 subsidiary categories and three subcategories and the constant comparison of the original data, we were able to identify a core category for our research: “preservice physical education teachers’ social media perceptions”. This core category served to integrate all of the other categories, concepts, and labels that we had identified through the coding process, providing a comprehensive and holistic view of the research topic. The core category allowed us to develop a theoretical framework that captured the key themes and patterns in the data and provided a solid foundation for our analysis and interpretation of the results.

In summary, the data collected for this study were rich and diverse, including interviews, literature, and social media posts. These data were labeled, conceptualized, and categorized through the open coding process. This allowed us to identify key themes and patterns in the data and to group the categories into subcategories through axial coding. Finally, through selective coding, we were able to develop a comprehensive theoretical framework for our research, consisting of a core category (“preservice physical education teachers’ social media perceptions”), three subcategories (Overall Perception, Value Perception, and Risk Perception), 10 subsidiary categories, 70 concepts, and 307 labels. This theoretical framework provided a solid foundation for our analysis and interpretation of the data and allowed us to better understand the complex and nuanced nature of preservice physical education teachers’ perceptions of social media.

In conclusion, the data collected for this study were initially conceptualized, then deeply conceptualized and categorized, and finally integrated into a theoretical framework through the grounded theory analysis process. This allowed us to identify key themes and patterns in the data and to develop a comprehensive understanding of preservice physical education teachers’ perceptions of social media. The resulting theoretical model provides a solid foundation for future research on this topic and offers insights into the potential challenges and benefits of using social media in physical education teacher education.

### 2.4. Trustworthiness

Guided by grounded theory, theoretical saturation is related to trustworthiness [40]. To ensure the reliability and validity of our coding process, we performed several tests and checks. This included conducting a coding consistency test to reduce subjectivity and increase confidence in the coding results. For this test, we selected five profiles at random from the original data and asked two researcher assistants who were familiar with the use of Nvivo 12 software to simultaneously code the sample back-to-back. We then calculated the coding repetition rate for the sample and compared it to the results obtained from the initial coding. Additionally, we conducted a theoretical saturation test using the remaining five interview profiles as reference materials. This allowed us to explore whether new categories and relationships could be identified from these profiles and whether the theoretical model constructed for the study had reached theoretical saturation. Overall, these tests and checks ensured the reliability and validity of our coding process and the resulting theoretical framework.

## 3. Results

**Figure 1:**
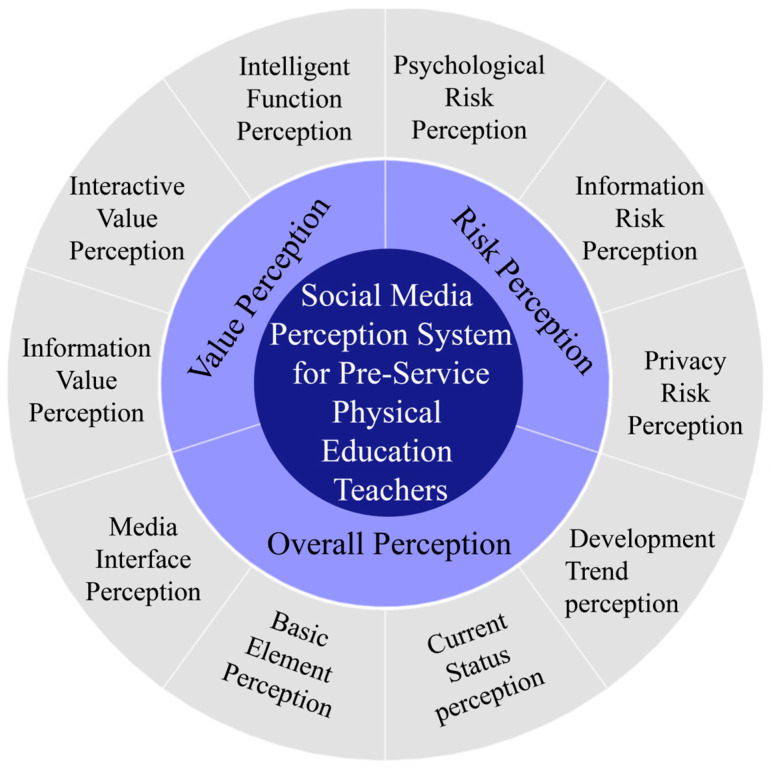
Pre-service physical education teachers’ perception of social media.

Through our analysis of the data, we identified three major themes that capture preservice physical education teachers’ perceptions of social media: (1) Value Perception, (2) Risk Perception, and (3) Overall Perception. Each of these themes represents a different aspect of preservice physical education teachers’ perceptions, and together they provide a comprehensive understanding of the complexities and nuances of these perceptions. Under each of these main themes, we identified numerous subthemes that further elaborated on the key concepts and categories identified in the data. These subthemes provided a more detailed and refined view of preservice physical education teachers’ perceptions of social media and helped us to better understand the factors that influence these perceptions (Figure 1).

### 3.1. Value Perception: Multiple Motivations for Social Media Usage

The theme of “Value Perception” refers to the reasons why preservice physical education teachers use social media and the expectations they have for its usage. This theme included subsidiary categories such as “Information Value Perception”, “Interactive Value Perception”, and “Intelligent Function Perception”. These categories captured the key factors that influence preservice physical education teachers’ decisions to use social media, as well as the benefits and opportunities that they expect to gain from its usage. Overall, the theme of Value Perception provided insight into the motivations and expectations of preservice physical education teachers in relation to social media.

#### 3.1.1. Information Value Perception

One of the most frequent subthemes under the theme of Value Perception was “Information Value Perception”. This subtheme referred to the value that preservice physical education teachers place on the wealth of information and resources available on social media. For example, many participants mentioned that social media provides them with access to a wide range of educational materials and resources that they can use to enhance their teaching practices. They also mentioned that social media allows them to connect with other teachers and experts in the field, which provide valuable insights and support for their professional development. Overall, the subtheme of Information Value Perception highlighted the importance of social media as a source of information and knowledge for preservice physical education teachers:

*The professional knowledge we can learn in school is relatively comprehensive and systematic. However, to some extent, it is not always what we want to learn, hence things we learnt in school are conventional and not very practical.*

(Participant 2)

One of the benefits of using social media for preservice physical education teachers is that it allows them to access cutting-edge knowledge and specialized information that is not readily available in traditional classroom settings. Through their interactions on social media, preservice physical education teachers may become aware of their own limitations and gaps in knowledge, which can motivate them to learn and grow as professionals. For example, some participants mentioned that they were able to learn about new teaching strategies, research findings, and trends in the field of physical education through social media, which helped them to improve their teaching practices and stay up-to-date with the latest developments in their field. Overall, the subtheme of Information Value Perception highlighted the potential of social media as a tool for professional growth and development among preservice physical education teachers.

*If we cannot update our knowledge and physical education philosophy in a timely manner, we are likely to fail to engage students in physical education, resulting in students increasingly finding physical education classes boring, as they can search for such knowledge content without the need for a teacher, and it is likely that the knowledge taught is what students have learned long ago.*

(Participant 3)

Overall, the theme of Value Perception revealed that preservice physical education teachers place a high value on the wealth of information and resources available on social media. This is particularly true for those who are motivated to learn and grow as professionals and who see social media as a valuable tool for accessing knowledge and expertise in their field. In this respect, the availability of rich and diverse information on social media emerged as the most significant consideration for preservice physical education teachers in relation to their use of social media.

#### 3.1.2. Interactive Value Perception

Participants also discussed the interaction as an enabler for social media usage. Regarding interactive perception, social media utilizing social networking technologies provide new opportunities for initiating “webs of enhanced practice” [42], where teachers around the globe can engage in collegial collaborations that enhance motivation for professional development.

Another key subtheme under the theme of Value Perception was “Interactive Value Perception”. This subtheme referred to the value that preservice physical education teachers place on the social and interactive aspects of social media. For example, many participants mentioned that social media provides them with access to online communities of fellow teachers and educators, which can serve as a valuable source of support, collaboration, and networking. These online communities can provide a sense of connection and belonging that may not be available in the offline world and can help preservice physical education teachers to feel less isolated and more engaged in their profession. Additionally, some participants mentioned that social media provides them with opportunities to connect with peers and experts from different parts of the world, which can broaden their perspectives and enrich their learning experiences. Overall, the subtheme of Interactive Value Perception highlighted the importance of social media as a platform for building community and fostering collaboration among preservice physical education teachers.

*I do feel that social media is very widely used nowadays, but in fact there is a big difference in the use of each person’s application. For example, everyone in our dormitory knows about Shake Voice, but several other students spend most of their time searching for some meaningless funny videos and amusing each other with the so-called stems, while I think that as a student most of my time should be spent on studying. So I spend most of my time on social media sharing videos I’ve learnt about jumping rope, and I’ve also joined a book sharing group to warn myself not to be complicit and to spend more time studying outside of class.*

(Participant 1)

#### 3.1.3. Intelligent Function Perception

Participants also highlighted Intelligent Function Perception. The subtheme of Intelligent Function illustrates the relationship between technology and social media. Digital technology is regarded as an extension of self, and social media is a primary mode of communication and social engagement [43].

Another subtheme under the theme of Value Perception was “Intelligent Function Perception”. This subtheme referred to the value that preservice physical education teachers place on the advanced technology and features that are integrated into social media platforms. For example, many participants mentioned that social media platforms include a range of intelligent functions, such as language translation, automatic sourcing of posts, and information storage and management, which can enhance their social media experience and make it more efficient and effective. Additionally, participants mentioned that the use of mobile devices and other technologies has made it easier and more convenient to access social media and that the well-rounded functions of these platforms fully meet their needs for professional development and communication. Overall, the subtheme of Intelligent Function Perception highlighted the importance of technology and advanced features in shaping preservice physical education teachers’ perceptions of social media.

### 3.2. Risk Perception: Concerns for Its Safety and Effectiveness

Despite reporting the perception of values that preservice physical education teachers perceive in social media, they also reported concerns about the risks and challenges associated with its use. The theme of “Risk Perception” captured the potential hazards and drawbacks that preservice physical education teachers perceive in their use of social media. This theme included three subsidiary categories: “Psychological Risk Perception”, “Information Risk Perception”, and “Privacy Risk Perception”. These categories captured the key factors that influence preservice physical education teachers’ perceptions of the risks associated with social media, as well as the potential consequences of these risks. Overall, the theme of Risk Perception provided insight into the challenges and concerns that preservice physical education teachers face in relation to their use of social media.

#### 3.2.1. Psychological Risk Perception

Participants expressed concern about the psychological risks associated with perception on social media. They noted that online posts on social media can lead to a phenomenon known as “context collapse,” in which a small mistake can cause significant changes to an individual’s identity in real life. This can be especially damaging for those who rely heavily on their online presence for personal and professional interactions:

*To maintain a professional image as a teacher in social media, we always need to be careful about what we say and do. Especially sometimes it might have a negative impact on the reality life (e.g., unemployed, accusation, damage the image of teachers or local school). As a result, I tend to reduce unnecessary social media interactions for avoiding misunderstandings.*

(Participant 6)

#### 3.2.2. Information Risk Perception

The final subtheme that emerged from the data was that preservice physical education teachers shared concerns about the limitations of information resources, which they referred to as “Information Risk Perception.” In this subtheme, participants frequently discussed concerns about the accuracy and professionalism of social media posts. They expressed a lack of trust in the information found on social media and the potential consequences of relying on such sources for professional development and decision-making. Some participants suggested that professional organizations and institutions should provide more reliable and systematic sources of information to help address this issue.

*Sometimes I feel overwhelmed about the posts related to physical education or sports, I can’t identify if it is true or not. For example, I found conflicting explanations of physical skill training methods in different sports social media platforms. So what do you expect this information? Trust or not? It is hard to say.*

(Participant 10)

*People send sports-related posts in social media are always not very serious, the same time, I find it difficult to screen the authenticity of basic sports theory or specific sports data in social media. So, I prefer to ask my teachers at university, it’s time consuming to find sports-related resources that fits my specific questions in social media.*

(Participant 12)

#### 3.2.3. Privacy Risk Perception

As the popularity of social media platforms such as WeChat and Micro-blog continues to grow, our privacy has become increasingly vulnerable to surveillance and even commodification. As preservice physical education teachers upload personal information to social media, more and more users are able to access this rich data at will. However, there is a lack of transparency and control over how this data is used and by whom, which can create a sense of anxiety and distress for preservice physical education teachers who use social media for professional development. This concern highlights the need for greater awareness and caution when using social media for professional purposes:

*It is true that there are a lot of people sharing physical education knowledge and skills on social media, and although we are also physical education students and are still relatively more knowledgeable about physical education than other users, because we are students at X school, we are expected to represent the school in our words and actions, and as a teacher educator, we are also expected to take an exemplary role and should not make random comments.*

(Participant 7)

### 3.3. Overall Perception: Uniqueness of Social Media for Teachers

Overall Perception refers to the general understanding of preservice physical education teachers’ perception of social media. This overarching view shapes their decisions on whether to use social media and for what purposes. Participants shared their imaginations of what social media looks like, and these imaginations were clustered into four subthemes: (i) media interface perception, (ii) basic element perception, (iii) current status perception, and (iv) development trend perception. These subthemes provide insight into preservice physical education teachers’ attitudes and beliefs about social media and its role in their professional development.

#### 3.3.1. Media Interface Perception

The definition of “media interface perception” is how users obtain and process information through the interface of social media platforms. This was also the initial impression that preservice physical education teachers had of social media before deciding to use it for professional development. In terms of media interface perception, the prevalent discussion among participants related to perceiving the quality of images and length of text on social media:

*For our physical education teachers, video is essential. Learn sports skills always related movement, so I prefer to search videos more than e-books. In social media, graphic videos related to sports or teaching practices always have high definition picture quality, clear and easy to understand.*

(Participant 11)

*We physical education teaching should be creative, so sometimes I like have unique design for my own social media page. Social media fits my need, I think the vivid and colorful interface matches the aesthetics of all pre-service physical education teachers.*

(Participant 14)

#### 3.3.2. Basic Element Perception

The subtheme of “basic element perception” highlights the concerns that preservice physical education teachers have about using social media for professional development. This subtheme includes their concerns about the financial cost and time investment required to access high-quality knowledge in physical education, as well as the information available on social media:

*As for the curriculum in universities, we are hardly find extra time to learn knowledge and skills comprehensively. Social media is such a time-saving way for us because I find it very easy to operate the video clips and other applications needed to share motor skills.*

(Participant 15)

*Buying a textbook or specialised books on sports disciplines are always expensive for our students, while I feel that paid sports resources in social media are affordable. But sometime the resources might be others posts by taking picture or recorded video with limited shooting angles. Such posts might affect the learning effect of technical movements.*

(Participant 9)

#### 3.3.3. Current Status Perception

The subtheme of “current status perception” reveals the participants’ views on the popularity of social media in university and outside of the university, both online and offline. This subtheme suggests that preservice physical education teachers recognize the widespread use of social media in their personal and professional lives and may be considering how to integrate these platforms into their professional development:

*There is no doubt that social media is a platform that welcome to everyone. We physical education teachers are such a group. While I still found physical education majors and teachers are using social media all the world!*

(Participant 2)

*Since I find that there are many users and even many authoritative physical education experts in social media. Those people are all interested in exercise and fitness, I think social media has solved exactly the paradox of learning and training that many pre-service physical education teachers faced.*

(Participant 5)

#### 3.3.4. Development Trend Perception

Participants also reflected on the future of social media and its potential impact on the field of physical education. Some participants mentioned that it may take time for new technologies to be fully integrated into regular physical education teaching in schools. Additionally, there were many participants who expressed ambivalent views about the use of social media in physical education, suggesting that there is still some uncertainty about the role of these platforms in the field:

*I feel (pause for almost a minute) it’s hard to say, for myself of course I think it’s good that social media is used in the classroom to save a lot of time in lesson preparation and students are exposed to a very rich knowledge of physical education, not just learning motor skills, but especially to make students aware of the value of physical education. But because physical education is given limited attention in schools, if schools were to make IT universal it would require a huge investment and could also cause opposition from parents. If that day does come then schools will most likely give the opportunity to subjects such as languages, maths, and foreign languages first, and it will take time for physical education lessons (social media applications) to become widespread.*

(Participant 11)

## 4. Discussion

According to the data collected from 17 preservice physical education teachers from various locations and types of universities in China, the present study investigated preservice physical education teachers’ perception of social media usage by uniquely employing Nvivo 12 software and grounded theory. Our findings answered three questions: (a) What aspects of social media do preservice physical education teachers find valuable? (b) What do preservice physical education teachers perceive the characters and elements of social media? (c) What kind of challenges have preservice physical education teachers faced in social media usage towards their professional development? By answering these questions, these reflections highlight the need for further research and discussion about the potential benefits and drawbacks of using social media for professional development in physical education.

### 4.1. Complex and Multi-Faceted Relationships among all Three Categories of Perception

The interplay between Overall Perception, Value Perception, and Risk Perception in social media is a complex and multi-layered phenomenon, as demonstrated below.

The relationship between Overall Perception and Value Perception in social media can be significant. Overall Perception, as the general understanding of preservice physical education teachers’ view of social media, can influence their Value Perception of social media. For instance, if preservice physical education teachers have a positive Overall Perception of social media, they may view it as valuable in terms of helping them stay connected with peers in their physical education teacher education program, and they may view the benefits of social media as increased social connectedness and reduced feelings of isolation. In contrast, if they have an overall negative perception of social media, they may view it as having limited value and see the risks as outweighing the benefits. Similarly, the relationship between Overall Perception and Risk Perception in social media is also important. Participants’ Overall Perception of social media can impact their views on the risks associated with its use. If preservice physical education teachers have a positive Overall Perception of social media, they may be less likely to view the risks as significant and may be more willing to continue using social media. Conversely, if they have an overall negative perception of social media, they may view the risks as more substantial and may be less likely to use it. In conclusion, the Overall Perception of preservice physical education teachers plays a critical role in shaping their Value Perception and Risk Perception of social media. A positive Overall Perception can lead to positive value and Risk Perceptions, while an overall negative perception can lead to a negative value and Risk Perceptions.

The Value Perception of social media can also impact views on its associated risks. If preservice physical education teachers believe that social media has a positive impact on their future careers, they may be inclined to view the risks as insignificant or manageable. Conversely, if they view social media as having limited value or if the risks outweigh the benefits, they may perceive the risks of social media use as more substantial. Similarly, Risk Perception of social media can affect how preservice physical education teachers engage with it. If someone believes that social media poses a significant danger to their privacy or well-being, they may reduce their usage or be more cautious when interacting with others in the field of physical education. For example, physical education teachers can post videos of exercises and activities, while health organizations can share infographics and illustrations to educate people on healthy habits. On the other hand, if they view the risks as manageable, they may continue to use social media without much thought to the potential risks. In the YouTube context, if preservice physical education teachers view instructional videos on physical education and health topics shared on YouTube as valuable, they may view the risks associated with using the platform, such as exposure to inappropriate content, as manageable and continue to use it for learning and professional development. Conversely, if they view the information shared on YouTube as less valuable, they may be less likely to use the platform or may view the risks as greater, potentially limiting their use of the platform.

In summary, the relationship between Overall Perception, Value Perception, and Risk Perception in social media is intricate and multi-faceted and can greatly impact how preservice physical education teachers use and engage with it. Preservice physical education teachers can learn that the way they perceive and engage with social media is influenced by their Overall Perception of it, as well as their perception of its value and the associated risks. They can use this information to carefully consider their use of social media, taking into account how it may impact their professional development and approach to teaching physical education. By being aware of the intricate and multi-faceted relationship between these factors, preservice physical education teachers can make informed decisions about using social media to advance their knowledge and skills and stay updated on the latest trends and developments in the field.

### 4.2. Social Media Reflects Preservice Physical Education Teachers’ Needs in a New Era

Our findings suggest that preservice physical education teachers perceive social media meets their professional needs in a new era.

Based on the theme of “Value Perception” in the discussions of preservice physical education teachers, it can be inferred that these teachers have a professional development need for gaining new information, opportunities for interactive communication and learning, and access to intelligent functions through the use of social media. These categories reflect the factors that drive their decision to use social media and the benefits they hope to gain from its usage. Thus, it can be concluded that preservice physical education teachers have a professional development need for exploring the potential of social media in advancing their knowledge, skills and overall professional growth. Based on the theme of “Value Perception” in the discussions of preservice physical education teachers, it can be inferred that these teachers have a professional development need for gaining new information, opportunities for interactive communication and learning, and access to intelligent functions through social media. These categories reflect the factors that drive their decision to use social media and the benefits they hope to gain from its usage. Thus, it can be concluded that preservice physical education teachers have a professional development need for exploring the potential of social media in advancing their knowledge, skills and overall professional growth.

Based on the Risk Perception we explored in this study, it can be inferred that the professional development needs among preservice physical education teachers include understanding and managing the potential risks associated with social media usage. This includes managing psychological risks, understanding the risks related to information, and managing privacy risks. One of the primary concerns identified is the understanding and management of potential risks associated with social media usage. This encompasses a wide range of potential risks, including psychological risks, information risks, and privacy risks. Regarding psychological risks, preservice physical education teachers need to be aware of the impact that social media can have on their well-being, such as the potential for cyberbullying, exposure to negativity, and the impact of social comparison. These teachers must also be equipped with strategies to manage these risks, such as setting healthy boundaries, seeking support, and engaging in self-care practices. In terms of information risks, preservice physical education teachers must understand the potential for misinformation, propaganda, and other forms of false information to be spread via social media. They need to be equipped with the skills to identify and verify the information and understand the importance of source evaluation. Finally, privacy risks are also a major concern for preservice physical education teachers. They must understand the potential for personal information to be shared or leaked on social media, as well as the dangers of oversharing personal information. They need to be equipped with strategies for managing their privacy, such as understanding privacy settings and being mindful of the information they share.

Based on the Overall Perception explored in our study, it can be inferred that the professional needs among preservice physical education teachers include a comprehensive understanding of social media and its role in their professional development. The Overall Perception of social media among preservice physical education teachers shapes their decisions on whether to use it and for what purposes. The study revealed that preservice physical education teachers have concerns about the financial cost and time investment required to access high-quality knowledge in physical education through social media, as well as the quality of information available on these platforms. The sub-themes of media interface perception and basic element perception provide insight into preservice physical education teachers’ attitudes and beliefs about social media. The media interface perception refers to how users obtain and process information through the interface of social media platforms and the initial impression that preservice physical education teachers have of social media. The basic element of perception highlights the concerns that preservice physical education teachers have about using social media for professional development, including the financial cost and time investment required to access high-quality knowledge. Furthermore, the study revealed that participants had mixed views about the future of social media and its potential impact on the field of physical education. Some participants expressed uncertainty about the role of social media in physical education, suggesting that there is still some ambiguity about its use in the field.

### 4.3. Teachers’ Perception of Social Media Followed Philosophical Foundations

“Goffman’s theory of self-presentation”, “Bourdieu’s theory of social capital”, “Sartre’s existentialism”, and “Heidegger’s theory of shared Theories” fit the connotations of the perception model we generated. These theories provided ideas for the process of generating this perception model of social media among preservice physical education teachers:

Self-representation theory is the ‘action’ basis for the generation of pre-service physical education teachers’ social media perception. Self-representation theory can be applied to the three categories of perception (Overall Perception, Value Perception, and Risk Perception) in social media usage among preservice physical education teachers. The theory suggests that individuals use self-representation to create a self-image that they present to others in order to gain social validation. For example, applying this to the Value Perception, preservice physical education teachers may use social media to present a positive self-image as knowledgeable, up-to-date, and connected professionals in the field of physical education. They may also use it to gain information and new ideas for their professional development or to interact with other professionals in their field to build relationships and networks. In applying this to Risk Perception, preservice physical education teachers may be concerned about the potential negative impact that social media usage can have on their self-image. They may worry about psychological risks, such as exposure to negativity or bullying, information risks, such as the spread of misinformation, or privacy risks, such as the potential exposure of sensitive information. These concerns may lead them to limit their social media usage or be more cautious in how they present themselves online. Finally, applying this to the Overall Perception, preservice physical education teachers may see social media as a tool that can serve both their self-representation and professional development needs but also understand the potential risks associated with its use. They may weigh the benefits and drawbacks of social media usage in light of their personal goals and values to make informed decisions about their engagement with the platform.

Social capital theory is the ‘zeitgeist’ basis for the generation of preservice physical education teachers’ perception of social media. The “Value Perception” theme highlights the motivations and expectations of preservice physical education teachers for using social media for their promising professional development. According to social capital theory, social media can provide opportunities for individuals to gain valuable connections and information that can increase their overall social capital. In this case, the preservice physical education teachers use social media to gain access to information that is relevant to their profession, such as pedagogical content, health promotion and body image, which might enhance their future teaching practice. In contrast, the “Risk Perception” theme highlights the potential hazards and drawbacks that preservice physical education teachers perceive in their use of social media. According to social capital theory, the risks associated with social media use can reduce an individual’s overall social capital. In this case, the preservice physical education teachers may be concerned about the potential consequences of using social media, such as psychological harm in their relationship with classmates in the physical education teacher education program, privacy violations, and information security, which can negatively impact their professional development via social media. The “Overall Perception” theme encompasses both value and risk perceptions and provides insight into the complex and multi-faceted relationship between these two factors. According to social capital theory, the Overall Perception of social media use can greatly impact an individual’s ability to gain social capital. In this case, the preservice physical education teachers’ Overall Perception of social media will influence their decisions to use it and their expectations for its usage, which can shape their professional development outcomes.

Shared world theory is the ‘driving force’ behind the formation of social media perceptual structures for pre-service physical education teachers. According to Heidegger, our existence in the world is characterized by care and concern for others [44], further supporting the conclusion that social media helps to reduce teachers’ sense of isolation and increase a sense of belonging [45,46,47]. Heidegger’s theory of shared theories suggests that individuals construct their own understanding of reality through their interactions and experiences with others. In the context of the three perceptions among preservice physical education teachers (Value Perception, Risk Perception, and Overall Perception), this theory suggests that their perceptions of social media are shaped by their interactions and experiences with others who use it. For example, if a preservice physical education teacher has positive experiences and interactions with other teachers who use social media, they are likely to have a positive Overall Perception of it and see its value for their professional development. On the other hand, if they have negative experiences and interactions, they may perceive more risks associated with using social media and be less likely to see its value. Therefore, the theory of shared theories highlights the role of social interactions in shaping an individual’s perceptions of social media.

### 4.4. Limitations and Future Research

Overall, this study suggests that while social media has the potential to be a valuable tool for professional development in physical education, it is also influenced by a range of objective factors. Given the limited sample size of this study, it is recommended that future research incorporate a larger sample to validate and expand upon these findings. Moreover, future studies should explore the theoretical model of social media perception among diverse groups, including in-service physical education teachers in primary and secondary schools, as well as university-based physical education teacher educators. Such research would yield a more comprehensive understanding of the role of social media in physical education and inform the development of effective strategies for using these platforms in professional development.

## 5. Conclusions

The theoretical model of social media perception among preservice physical education teachers was explored using Grounded Theory, resulting in the generation of the core category called Social Media Perception System for Preservice Physical Education Teachers. The system contained three subsidiary categories, such as “Overall Perception”, “Value Perception”, and “Risk Perception”. Inside these subsidiary categories, there are different sub-categories inside, like “Overall Perception”, “Media Interface Perception”, “Basic Element Perception”, “Current Status Perception”, and “Development Trend Perception”. Value Perception includes “Information Value Perception”, “Interactive Value Perception”, and “Intelligent Function Perception”. Risk Perception includes “Psychological Risk Perception”, “Information Risk Perception”, and “Privacy Risk Perception”. The above 10 sub-categories and three subsidiary categories are generated based on 70 concepts and 307 labels. This systematic theory has passed the theoretical saturation test and coding consistency test, fully indicating it has good reliability.

The findings of this study on the social media perception among preservice physical education teachers can provide valuable insights into the level of understanding and use of these platforms among this group. Our findings can be used to identify the challenges and concerns that preservice physical education teachers have about using social media for professional development and inform the development of education programs and strategies for addressing these issues. Importantly, this study can help administrative departments to evaluate preservice physical education teachers’ information technology capabilities and determine how to support their use of social media for professional development. Specifically, preservice physical education teachers could benefit greatly from understanding the theme of “Value Perception” in social media. This theme refers to the reasons why they use social media and their expectations for its use, including the informational, communicative, and functional benefits. By comprehending these Value Perceptions, preservice physical education teachers can adopt a more thoughtful and strategic approach to using social media for their professional development. Moreover, by understanding the “Risk Perception” of social media, preservice physical education teachers can be aware of the potential hazards and drawbacks associated with its usage, including psychological, informational, and privacy risks. This awareness can help them to exercise caution in their online behavior and information sharing, as well as take measures to protect their privacy and security. Finally, by grasping their Overall Perception of social media, preservice physical education teachers can be more deliberate and purposeful in their usage, aligning it with their professional development goals while avoiding potential pitfalls.

In order to effectively support the professional development of preservice physical education teachers in the era of information technology, it is important to understand their perceptions of social media and how it can be used for professional development. This study provides valuable insights into the social media perceptions among preservice physical education teachers, but it is recommended that future research be conducted to validate and expand upon these findings. By examining the social media perceptions of preservice physical education teachers in different regions, we can gain a more comprehensive understanding of the challenges and opportunities that these platforms present for professional development in physical education. This knowledge can inform the development of effective strategies for supporting preservice physical education teachers in their use of social media for professional development.

The grounded theory that we used for our study has benefits and also limitations. Grounded theory is a research innovation that holds the potential to greatly benefit the study of constructing Chinese preservice physical education teachers’ perception model of social media in several ways. Firstly, the inductive nature of grounded theory enables the development of a theory that emerges from the data rather than being predetermined by pre-existing theories or models. This approach is particularly advantageous in perception research as it allows the researcher to gain a deeper understanding of the unique perspectives, experiences, and behaviors of the participants in the specific context of Chinese preservice physical education teachers. Furthermore, the iterative process of data collection and analysis inherent in grounded theory enables the refinement of the researcher’s understanding of the participants’ perceptions as more data is collected. This approach results in a more nuanced and comprehensive understanding of the participants’ experiences and perspectives, which is critical in perception research, where the objective is to comprehend how individuals perceive and make sense of their experiences. However, there are also some potential limitations to using grounded theory in this study. One limitation is that grounded theory relies heavily on qualitative data, which can be subjective and open to interpretation. This may result in a limited understanding of the phenomenon being studied if the data is not collected and analyzed rigorously. Moreover, the grounded theory approach requires a significant investment of time and resources, as the researcher must collect, analyze, and interpret a large amount of data to develop a theory that is grounded in the participants’ experiences. This can be a challenge, particularly in cross-cultural research, where there may be language barriers or cultural differences that affect the data collection and analysis process. In conclusion, while grounded theory offers several benefits for the study of Chinese preservice physical education teachers’ perception of social media, there are also potential limitations that must be considered. The researcher must carefully consider these limitations and ensure that the data collection and analysis methods are rigorous and appropriate for the research question being addressed.

## Figures and Tables

**Table 1:** Demographics for the sample of participants (N = 17).

No.	Gender	Class	Parts	Type of University	Usage Duration (Years)	Usage Frequency (h/per Day)
1	Female	Senior	North-East	Normal College	Five	3–5
2	Male	Junior	South-West	Normal College	Seven	6–8
3	Female	Junior	South-West	Normal College	10	6–8
4	Male	Senior	North-East	Normal College	11	3–5
5	Female	Senior	South-West	Normal College	13	3–5
6	Male	Senior	North-East	Normal College	Eight	3–5
7	Female	Freshman	South-East	Normal College	14	6–8
8	Female	Senior	North-West	Comprehensive University	10	6–8
9	Female	Senior	South-East	Comprehensive University	13	3–5
10	Male	Senior	North-East	Comprehensive University	12	3–5
11	Female	Sophomore	South-East	Comprehensive University	Nine	6–8
12	Male	Junior	South-East	Comprehensive University	Eight	3–5
13	Male	Junior	South-East	Comprehensive University	Seven	9–11
14	Male	Senior	South-West	Comprehensive University	10	6–8
15	Male	Senior	South-East	Sports College	11	3–5
16	Male	Junior	South-East	Sports College	13	6–8
17	Female	Senior	North-East	Sports College	14	9–11

**Table 2:** Interview Questions.

No.	Interview Questions
1	How do you define the elements of social media and what role you see it playing in your future career as a physical education teacher?
2	What do you think the future trending of using social media in your future job as a physical education teachers? Talk about the ideas and its reasons.
3	What is the advantages and disadvantages of using social media for your professional development in physical education? Talk about it with your social media usage experience.
4	How do you evaluate the credibility of information found on social media in regards to physical education and health?
5	Have you ever encountered false information on social media related to physical education or health? How did you handle the situation?
6	What experience makes you do not want to use social media for your professional development journey as a physical education teacher?

**Table 3:** Labeling results.

Tagged Labels	Tagged Labels
E1 I feel it is necessary to use social media for motor skill learning	E155 It is difficult to share the physical education knowledge and motor skills learned in class on social media
E2 I find that sports-related learning resources in social media are cutting edge	E156 Unclear how to demonstrate preservice physical education teachers’ professionalism in social media
…….	
E152 Social media is good for maintaining the habit of learning something new every day	E306 It is necessary to ask other users in social media for knowledge and methods of physical and health education
E153 The need to proactively plan application time and learning content in the application of social media	E307 The need to reflect on and master the means of encouraging student communication in social media communication
E154 Social media is very informative about the knowledge needed to plan sports events	

Notes: Reported codes are the conceptual labels applied to the transcribed data. For specific data, see Table A1.

**Table 4:** Conceptualization results of 307 tagged labels.

Number	Conceptualization Concepts	Tagged Labels	Number of Materials	Number of Participants
D1	I find the graphic videos related to sports or teaching practices on social media smooth, clear and easy to understand	4 articles: E297, E299, E302, E154	54	5
D2	I think there is a lack of communication boards for issues related to physical education	1 Article: E33	3	2
D3	I think pre-service physical education teachers are happy to share or exchange motor knowledge skills in social media	6 articles: E272, E261, E83, E170, E3, E87	21	4
………..				
	Total	307 articles	1122	22

Notes: Articles are the results from the first coding; the participants mean the people referred to the code and the materials mean the interview, online posts and literature referred to the code. For specific data, see Table A2.

**Table 5:** Results of categorization of 70 concepts.

Subsidiary Categories	Concepts
	D1 I find graphic videos related to sports or teaching practices in social media smooth, clear and easy to understand
C1	D13 I feel that the typographic interface of social media is clean and clear for pre-service physical education teachers
Interface Perception (4)	D55 I think the vivid and colorful interface matches the aesthetics of all pre-service physical education teachers
	D25 I believe that the vehicles of videos, photographs, captions, and others are very suitable for the overall development of pre-service physical education teachers’ professional perceptions
……	
C10	D63 Social media messages from non-sports professionals make me feel very culturally different
Psychological Risk (8)	D38 I feel it is difficult to receive timely responses from high-level physical education teachers or research experts in social media
	D52 Need to have the self-discipline needed to concentrate on learning about sports in social media
	D54 I feel that most of the active sports users are motivated by the intention of selling skills courses or nutritional supplements, etc.
	D69 I feel that the sharing of novel exercise skills in social media is more engaging than in offline classes but it is easy to get distracted

Notes: For specific data, see Table A3.

**Table 6:** Results of the main axis coding for 10 categories.

Sub-Categories	Subsidiary Categories
B1 Overall Perception	C1 Interface Perception
	C2 Element Perception
	C3 Current Status Perception
	C4 Development Trend Perception
B2 Value Perception	C5 Information Value Perception
	C6 Interactive Value Perception
	C7 Intelligent Function Perception
B3 Risk Perception	C8 Psychological Risk Perception
	C9 Privacy Risk Perception
	C10 Information Risk Perception

## Data Availability

Data can be shared upon reasonable request to the corresponding author.

## Appendix A

**Table A1:** Labeling results.

Tagged Labels	Tagged Labels
E1 I feel it is necessary to use social media for motor skill learning	E155 It is difficult to share the physical education knowledge and motor skills learned in class on social media
E2 I find that sports-related learning resources in social media are cutting edge	E156 Unclear how to demonstrate preservice physical education teachers’ professionalism in social media
E3 I think non-sports professional sports fans should be respected in social media	E157 Asking front-line physical education teachers for advice in social media does not receive a response
E4 Unscientific sports knowledge skills shared by users in social media	E158 I understand that it takes time to receive responses from physical education experts, frontline physical education teachers
E5 Multiple social media platforms should be used to learn motor skills	E159 Should respond to other users’ questions about physical activity in a timely manner on social media
E6 Recognize that there is a lot of fitness and fat loss knowledge shared in social media	E160 What is learned in the physical education classroom should be shared generously on social media
E7 Mastery or in-depth understanding of specific sports skills (e.g., basketball) is easy on social media	E161 I feel that the information of sports in social media is updated relatively quickly
E8 There should be no bias against non-sports professional users in social media	E162 Social media should be used to proactively update physical education teaching or learning methods
E9 Identify the mixed messages in social media and find the right resources for physical education learning	E163 Should actively strengthen the connection with fellow sports majors through social media
E10 I think social media for fragmented knowledge learning is good for balancing after-school training and physical education learning	E164 Recognize the lack of clarity in the division between motor skills and sport theory in social media
E11 Should use social media to improve their sports knowledge and skill deficiencies	E165 The need to use social media to enhance one’s sport-specific learning
E12 I think it is important to maintain good ethics when making statements on social media	E166 I feel that the graphical form of knowledge sharing in social media is interesting
E13 I believe that exercise questions from users of different skill levels should be accommodated	E167 I feel I can master other disciplines through social media
E14 I feel that social media stimulates my interest in learning about other subjects	E168 I feel that I can urge myself to study sports through social media
E15 I have the confidence to share sports knowledge and skills on social media	E169 should use social media such as WeChat sports and KEEP to motivate themselves to develop physical fitness
E16 Interpretation of physical education policies related to the presence of frontiers in social media	E170 I feel able to reach frontline physical education teachers very easily
E17 I think information is updated very quickly on social media	E171 I think it is important to focus on the question of the existence of different types of users of sports learning in social media
E18 I feel that the learning effect in social media is easily affected by noise interference	E172 I feel that I get rich PE learning resources through social media such as Jitterbug, WeChat, and Zhihu
E19 Scientific integration of fragmented sport theoretical knowledge in social media is more difficult	E173 should be generous in communicating with non-physical education users
E20 I feel that the chances of receiving a response on social media are small	E174 I find it very easy to master social media sharing, commenting, etc.
E21 I feel that there is more knowledge and methods related to teaching health in social media	E175 I found that there are many users who are concerned about sports nutrition, fitness, and fat loss
E22 Recognize that the future of sports networking resources in social media will be increasingly rich and complete	E176 Active participation in social media discussions on anonymous topics is essential
E23 Recognize that sharing motor skills in social media requires some editing skills	E177 Physical education values should be boldly shared on social media
E24 We should selectively search for content in social media based on goals	E178 Clearly communicating sports values in social media is easy
E25 You can learn things in social media that you can’t learn in offline sports classes	E179 It’s easy to search through social media to find the information you need for your sports internship
E26 A user in social media shares tips for writing a sports thesis	E180 I am concerned about the security risks of identity data in social media
E27 finds that smart pushing is likely to distract you	E181 Social media’s smart push feature should help with professional development
E28 Knowledge content shared by users of different motor skill levels should be included	E182 Should proactively engage in learning exchanges with teachers of other disciplines in social media
E29 Social media on different platforms should be used to test the correctness and effectiveness of learning physical education knowledge skills	E183 The professional, ethical qualities required of pre-service physical education teachers should be observed in social media
E30 Recognize social media as a complementary approach to formal pre-service physical education	E184 Scientific health and fitness tools and techniques should be mastered through fun videos in social media
E31 Social media facilitates communication with surrounding physical education teachers	E185 You can learn what you need for your future work as a physical education teacher through the push of authoritative information
E32 Enhance the understanding of the techniques and tactics of each event through the video replay function in social media	E186 Facilitating the development of a full range of specialized motor skills through social media
E33 Social media’s smart push feature facilitates holistic learning	E187 Learning in Social Media Requires Active Reflection and Summarization
E34 The process of retrieving and gathering useful sports information is rapid	E188 I have a psychological burden to initiate contact with in-service physical education teachers among unfamiliar users
E35 I feel able to update my understanding of the organization of teaching and learning through the sharing of physical education teachers	E189 Mastering basic functional modules such as social media sharing and commenting is easy
E36 I feel that the influence of the national context and educational system on physical education is shaped through authoritative information in social media	E190 Able to learn the physical education and health curriculum model through Zhihu, WeChat, etc.
E37 Physical games and situations that increase motor load should be created through public sharing content	E191 Social media enables understanding of the future direction of physical education
E38 Language translation function can facilitate the understanding of foreign physical education teachers	E192 Understanding of physical education curriculum development should be deepened based on cutting-edge information from social media
E39 Social media enables me to master rich evaluation content and diverse evaluation methods	E193 Clear and accurate communication of sports values in social media is a responsibility
E40 Should think independently about the content of information in social media	E194 Recognizing that social media is beneficial in breaking down the inequitable distribution of physical education resources
E41 Should learn from the content shared by frontline physical education teachers to adhere to the concept of innovative curriculum reform	E195 Should actively participate in group seminars in social media
E42 Should take the initiative in social media to learn the knowledge and skills needed for future teaching and learning	E196 Social media should be used for the promotion of physical education
E43 Learning activities in social media require a high level of self-awareness	E197 Entertainment and irrelevant information in social media should be actively avoided
E44 Social media should be used for active learning of physical education and health curriculum standards	E198 Attempts should be made to openly share knowledge and skills learned in physical education in social media
E45 Social media should be used to transform student teachers’ identities to facilitate adaptation to teaching physical education	E199 Should actively promote the value of physical activity in social media to contribute to sports for all
E46 You can learn about the knowledge and skill requirements for physical education teacher recruitment in different regions	E200 Recognize that GIFs in social media are conducive to movement representations
E47 Should use social media to meet fellow students with the same sport	E201 It is difficult to find the right exercise exercise for yourself in social media
E48 Presence of knowledgeable information to correctly judge exercise load metrics in the physical education classroom	E202 Be able to learn about the basic tools of sports rehabilitation in social media
E49 It is easy to search for suitable training programs based on keywords in social media	E203 It is helpful to use social media to communicate with talented scholars from other disciplines
E50 Should actively study the interpretation of physical education curriculum reform shared by authoritative users	E204 Applying physical education knowledge or skills learned from social media in the physical education classroom is difficult
E51 Difficulties in understanding users’ interpretations of the Chinese health and physical education curriculum model	E205 Positive sports values learned through social media should be shared with classmates
E52 Social media should be used to actively learn the basic knowledge and skills of sports competition and officiating	E206 Should be proactive in accessing physical education resources outside the classroom on social media
E53 Should reflect on and review the knowledge and skills learned about sport on social media	E207 Should confidently engage in academic sport communication with other users on social media
E54 Should maintain an active learning mindset about the physical education skills shared by classmates	E208 Questions that arise in the physical education classroom should be answered on social media
E55 Active communication with teachers of other disciplines through social media can enhance interdisciplinary foundational knowledge (language, math, English, etc.)	E209 Should communicate with unfamiliar users on social media in a friendly manner and take the initiative to ask for help when encountering problems
E56 Sports knowledge and skills should be actively shared in social media group chats	E210 Should proactively counteract stigmatizing statements about physical education teachers in social media
E57 Physical education curriculum resources in social media can be used to deepen the understanding of physical education	E211 Social media should be used to proactively update sport knowledge and skills
E58 should demonstrate sportsmanlike qualities during communication with other users on social media	E212 Emoticons can demonstrate the humorous qualities of pre-service physical education teachers
E59 Physical education learning in social media as a reflection of professional development concepts that foster lifelong learning	E213 Related physical education methods that can be learned in social media to create game situations
E60 Feeling that experts and scholars generally do not respond to messages	E214 Collaborative learning with other pre-service physical education teachers should be developed in social media
E61 The ability to motivate other users to be physically active through social media	E215 Be able to master specific and feasible methods of writing a thesis in physical education in social media
E62 Clear and strict hours and precautions for social media use should be established	E216 Knowledge and skills learned through social media should be shared with other pre-service physical education teachers through reproduction
E63 Should be active on social media to learn the advantages and disadvantages of pursuing a career as a physical education teacher	E217 Should proactively use social media to update their understanding of the physical education process
E64 The initiative to identify sports problems and actively reflect on them should be developed in independent learning on social media	E218 Should be polite and respectful in their interactions with other users on social media
E65 Being able to learn the main spirit of the new curriculum standards in social media	E219 Social media can be used to efficiently solve the problems faced in the physical education classroom
E66 Able to learn about common disease prevention tools in social media	E220 Personal interests can be understood through a wide range of physical education learning resources in social media
E67 It is difficult to reach out to coaches on social media about ways and means to conduct sports competition activities	E221 should discover the beauty of each sport through the sports videos shared by other users
E68 There is a wealth of knowledge and skills related to assembly and formation in social media	E222 Should actively participate in discussions about teaching presentations in social media
E69 A mindset of experimentation with teaching models shared by other users	E223 Should communicate with officially certified PE seniors on social media about PE curriculum reform
E70 Social media allows you to buy sports coaching courses at a good price	E224 Should learn about physical education through social media needed to develop curriculum resources
E71 Discussion of sports research through social media is common	E225 I feel that there are too many categories of information which makes it difficult to focus on the content related to my major
E72 It is difficult to make practical attempts at quality skill videos on social media in conjunction with one’s own physical fitness base	E226 Difficulty in clearly integrating and categorizing motor skills learning materials in social media
E73 can encourage other users to actively participate in exercise through likes, etc.	E227 Recognizing their own shortcomings in comparison with outstanding teachers in social media
E74 can encourage sports fans to enjoy a healthy life through private chat	E228 Possibility to discuss curriculum development with physical education teachers from other districts in social media
E75 should provide appropriate physical training methods for users of different ages	E229 Should share their enthusiasm for participating in sports on social media
E76 Knowledge sharing through social media can enhance the experience of teaching physical education	E230 Problems should be identified in the skill practice videos shared by other physical education teachers
E77 should be a combination of voice, video, pictures and other ways to provide physical exercise advice to other users	E231 You can learn the content of the school-based curriculum on social media
E78 Conflicts between social media learning and formal physical education classroom learning should be balanced	E232 Students around you should be encouraged to join social media to learn together
E79 Physical exercises to learn reasonable exercise load easily through voice calls	E233 You can objectively know your own sports skill level from other users’ evaluation of your own shared content
E80 Should take the initiative to update their knowledge base in their area of expertise by using the wide range of knowledge information in social media	E234 Outdated sports knowledge and skills in social media should be properly screened out
E81 Social media group discussions contribute to the concept of equality for all	E235 Fraudulent information in social media should be properly screened out
E82 Mastering information technology operational skills related to the live delivery of sports knowledge is simple	E236 Awareness of spreading physical culture should be developed in the sharing of physical exercise content
E83 Physical education learning in social media should be viewed correctly and still applied offline	E237 Should search social media for ways to teach students specific motor skills
E84 Discussions can be held with special education teachers on social media to address the learning disabilities of students with special needs	E238 Access to physical education teachers who have extensive experience in sports and games
E85 The source should be indicated in the reproduction of other people’s sports knowledge and skills in order to respect the fruits of their work	E239 Front-line physical education teachers in social media should be used to ask for advice on ways to master the orderly conduct of the classroom needed for the internship
E86 It is necessary to take on the mission of passing on the spirit of sports in the content that you share	E240 Should be proactive in providing physical skills instruction to sports enthusiasts in social media to the best of their ability
E87 Should share the knowledge of motor anatomy learned in social media with classmates in offline classes	E241 Should actively participate in discussions on topics related to physical education materials in social media
E88 Mental health teachers in social media should be used to ask for tips on how to self-direct negative emotions and counsel students	E242 Be inspired by other users’ sharing of physical education approaches and methods
E89 Should be proactive in learning about sports and health-related content on social media	E243 should take the initiative in social media to learn teaching methods that are appropriate for students of different levels
E90 Physical activity videos shared on social media can improve physical expression	E244 In social media, you can give your opinion about the new curriculum reform
E91 Sports skill exercise videos posted by non-sports professional users should be treated inclusively	E245 The required behaviors of pre-service physical education teachers should be maintained in social media communication
E92 Seeing students around me share sports knowledge frequently makes me feel pressured	E246 Should actively learn sports competition-related knowledge skills in social media
E93 Unfamiliar users in social media should be encouraged to participate in health-promoting campaigns	E247 The need to participate in free sports discussions in social media for training and further education
E94 Ability to learn the methods of physical education learning assessment needed to engage in physical education teaching in social media	E248 Should follow the new curriculum in social media
E95 Need to form the habit of applying social media to improve various knowledge skills	E249 Should reflect on the appropriate physical education teaching methods from the physical education teaching videos posted by others
E96 Issues related to curriculum reform should be discussed with pre-service physical education teachers in social media	E250 Misconceptions about sports among users in different industries should be viewed inclusively
E97 Ability to use variable speed replay in social media to repeatedly learn what was learned in a physical education class	E251 Should defend shared sports video rights in social media
E98 Should take the initiative to absorb new concepts of physical education according to the requirements of curriculum reform	E252 Identity for physical education curriculum reform should be expressed in social media communities
E99 The public should be used to learning cutting-edge international methods of teaching physical education	E253 Physical education strategies for students of different levels can be learned in social media
E100 should confidently share sports knowledge through live streaming on social media	E254 Discussing sports with other users in a good mood on social media is necessary
E101 Should respond to other users’ questions and opinions about sports in an inclusive and understanding manner	E255 Social media professional learning communities facilitate the formation of a pre-service physical education teacher identity
E102 Should proactively use social media for knowledge sharing	E256 Ability to learn new competencies from other industry users in social media
E103 Relevant users who should follow sports research papers on social media	E257 Stimulating interest in physical activity through content shared by other pre-service physical education teachers
E104 Physical education classroom management can be learned through social media	E258 Feeling that WeChat sports and Yuexing circle are good for developing healthy lifestyle habits
E105 Should actively search for relevant content about health education	E259 Ability to learn to write about research topics in social media
E106 Health education-related topics should be proactively discussed on social media	E260 Users related to traditional ethnic sports should be followed on social media
E107 Social media can facilitate the learning of sociological and psychological knowledge skills	E261 Should actively reach out to teachers of other disciplines in social media
E108 Social media facilitates the formation of a mission of lifelong learning	E262 It is necessary to discuss or ask for sports advice from other users in social media in a humble and polite manner
E109 Mastering and applying educational general education methods and techniques in social media is convenient	E263 It makes sense to provide sports nutrition-related knowledge to unfamiliar users
E110 The quick communication value of social media should be used to strengthen ties with neighboring physical education teachers	E264 Collaborative communication with teachers across disciplines in social media is feasible
E111 Discussions in various virtual communities in social media facilitate the collection of sports-related knowledge resources	E265 It is necessary to follow users related to sports rehabilitation and massage on social media
E112 Conversations on social media with non-sports professional users should be respectful	E266 The target audience of social media is becoming more and more popular
E113 Social media should be used to understand the goals of teaching based on different levels of standards	E267 Ability to learn about health care in social media
E114 Should be passionate about answering sports questions for others on social media	E268 The new curriculum standards published for different physical education majors share different content
E115 Searching for basketball three-step layups is too broad a technical move and feels impractical	E269 finds that the age range of users in social media is getting wider
E116 Join the live sports to get “dry” sports skills practice	E270 should share the cutting-edge sports resources learned with classmates on social media
E117 Include learning about sports character and moral development in social media	E271 should learn from other users’ excellent sports learning methods and reflect on their own shortcomings
E118 Videos reflecting on shared skills in social media facilitate the development of a unique teaching style	E272 Need for active dissemination of health promotion knowledge and skills in social media
E119 Rumors should be avoided in social media and wrong methods of physical exercise should be resisted firmly	E273 Proactive exchange of physical education issues with pre-service physical education teachers abroad is necessary
E120 The need for active learning in social media related to core literacy in physical education and health disciplines	E274 The use of social media is useful to assist in improving the technical level of exercise
E121 Should learn to create sports and health-related community teams on social media	E275 Discover the increasingly diverse composition of users in social media
E122 Being able to learn the regulations of physical education and its models in developed areas in social media	E276 Should consciously focus on positive sports knowledge content in social media
E123 Sharing health knowledge skills posted on social media should be of interest to other users	E277 Most of the content should be posted by non-sports professional users
E124 Should develop good study habits in social media (e.g., self-directed learning)	E278 Should consciously learn the smart push model of Chinese health physical education curriculum
E125 Physical education policies in social media are conducive to renewed parenting models	E279 Social media sharing facilitates the advancement of one’s physical education skills
E126 Learning skills related to improving a certain type of sports training but often find irrelevant content	E280 Social media applications should be seen as a means of professional development and should be planned and rigorously implemented
E127 Methodological skills necessary to share health knowledge and competencies in social media	E281 finds more and more sports users on social media
E128 By speaking positively in the group chat should be the principle of positive energy	E282 Active reflection should be made from the communication with the physical education needs of users of different ages to develop a student-led concept of physical education
E129 Exercise routines should be shared to demonstrate the sunshine passion of pre-service physical education teachers	E283 Share successful physical education learning methods with preservice physical education teachers in social media
E130 Users with local teaching characteristics should be followed in social media to reflect on ways to create a quality physical education	E284 Should take the initiative to learn the official information of social media and try to improve their original cultural base
E131 Asking experienced physical education teachers in social media for advice on effectively handling conflict among students is effective	E285 Should proactively seek advice from frontline physical education teachers on strategies and methods for leading sports club activities
E132 Need to have the aesthetic sensibility to properly examine a wide range of information content on social media	E286 Should be active in social media to learn the concept of physical education and health curriculum reform
E133 should implement the physical education learning method of online learning and diligent offline practice	E287 The professional development spirit of pre-service physical education teachers should be reflected in social media sharing
E134 Social media topic discussions reflect the concept of teacher community	E288 Feelings of accomplishment in providing physical activity assistance to others on social media
E135 Sports content in social media is conducive to increasing commitment and perception of the value of physical activity	E289 Information retrieval in social media should be aimed at forming an ideal for education
E136 In social media is conducive to learning the relevant systems promulgated by the Party and the State	E290 Feeling that it takes a lot of time to find professional physical education knowledge skills
E137 The need to use social media as a way and tool to address physical education classroom issues	E291 Ability to learn and master new sports in social media
E138 Understand the knowledge and skills of sports shared by others on social media	E292 should reflect on the methodological means of applying social media to physical education teaching and professional development
E139 Share sports exercise videos need video shooting and editing skills	E293 The need to use social media for continuous learning as a way to refine quality
E140 believes that sports videos shared on social media are all helpful to other users	E294 Sharing sport knowledge skills in social media as a pathway to professional development
E141 Recognize that students around them are joining social media	E295 Recognize that the use of social media is a major trend in education and teaching
E142 Recognize that social media can be used as a tool to hone their own educational and teaching skills	E296 Social media facilitates the development of ideas for continuous learning and application
E143 Share sports knowledge and sports skills needed by other users on social media	E297 Perceived that the content of information in social media has little knowledge of the physical education profession
E144 The content of physical education-related resources should be shared in an easy-to-understand manner	E298 Should be active in social media to learn the content of core literacy in physical education and health subjects
E145 Respect for all users must be maintained in social media communication interactions	E299 It is difficult to learn professional bandage rehabilitation techniques on social media
E146 Social media is good for learning about foreign movement patterns and discussing and communicating with pre-service physical education teachers around them	E300 Felt intimidated by the prospect of asking an authoritative front-line physical education teacher for advice on professional issues
E147 The need to maintain collective solidarity and collaboration in professional communities in social media	E301 Should demonstrate sports skills confidently on social media
E148 The need to confidently present pre-service physical education teachers in social media	E302 Hope there will be more sports-related exchanges and interactions in the future
E149 The need to focus on students’ athletic interests and needs in social media	E303 Should master and apply content knowledge in various forms, such as reproduction in social media
E150 Knowledge skills related to common sports injury prevention and control in social media	E304 A cutting-edge approach to teaching physical education in social media
E151 The process of social media application facilitates the development of a wide range of reading habits	E305 Recognizing the value of fitness in contemporary physical education in the context of social media
E152 The use of social media is good for maintaining the habit of learning something new every day	E306 It is necessary to ask other users in social media for knowledge and methods of physical and health education
E153 The need to proactively plan application time and learning content in the application of social media	E307 The need to reflect on and master the means of encouraging student communication in social media communication
E154 Social media is very informative about the knowledge needed to plan sports events	

**Table A2:** Conceptualization results of 307 tagged labels.

Number	Conceptualization Concepts	Tagged Labels	Number of Materials	Number of Participants
D1	I find the graphic videos related to sports or teaching practices on social media smooth, clear and easy to understand	4 articles: E297, E299, E302, E154	54	5
D2	I think there is a lack of communication boards for issues related to physical education	1 Article: E33	3	2
D3	I think pre-service physical education teachers are happy to share or exchange motor knowledge skills in social media	6 articles: E272, E261, E83, E170, E3, E87	21	4
D4	I find it difficult to screen the authenticity of sports-based theories or specific sports data on social media	3 articles: E4, E9, E33	5	3
D5	I believe that pre-service physical education teachers can demonstrate strong professional ethics in their interactions with users	6 articles: E12, E8, E156, E13, E183, E199	87	10
D6	I feel that the information about the sports skills practice category and the sports theory knowledge category is fragmented	9 articles: E153, E152, E151, E291, E303, E300, E298, E292, E136	14	3
D7	I think physical education majors and teachers are using social media	7 articles: E275, E31, E240, E145, E112, E91, E8	33	6
D8	I feel that pre-service physical education teachers who lack teaching experience are prone to misunderstandings or arguments during social media sharing	3 articles: E158, E60, E300	11	4
D9	I feel that there is a lack of authoritative sources of information from frontline physical education teachers, sports research experts, and official sports institutions	4 articles: E290, E297, E299, E277	10	4
D10	I feel that the division between motor skills and sports theory in social media is not clear	2 articles: E115, E126	9	2
D11	I think in the future, all social media will be interconnected to make it easier to access physical education resources.	1 Article: E136	4	2
D12	I feel that the vast amount of sports resources on social media has made school no longer the only gateway for pre-service physical education teachers to gain knowledge and skills	7 articles: E181, E59, E62, E280, E287, E294, E30	20	2
D13	I feel that for pre-service physical education teachers, the typographic interface of social media is clean and clear	2 articles: E23, E139	23	6
D14	I feel that social media has freed pre-service physical education teachers from the constraints of uniform offline content and teaching schedule	3 articles: E250, E15, E212	7	4
D15	I feel that social media will encourage the sharing of knowledge on sports nutrition, sports and fitness skills in the future	2 articles: E169, E22	12	4
D16	I found that pre-service physical education teachers were able to participate more actively in discussions about physical education topics in an anonymous setting	10 articles: E147, E182, E188, E203, E209, E67, E89, E105, E120, E286	4	1
D17	Limited camera angles for live or recorded video may affect the learning of technical movements	1 Article: E109	36	5
D18	I think the future of online sports course resources on social media will be more systematic and complete	2 articles: E301, E19	1	1
D19	I feel that in the future, knowledge about sports skills practice and sports theory will be more and more clearly classified	1 Article: E160	7	6
D20	I find the video clips and other applications needed to share motor skills very easy to operate	6 articles: E27, E85, E102, E147, E134, E262	8	2
D21	I think the video replay and shifting function make the dynamic changes in each item’s technique and tactics easier to understand	4 articles: E129, E254, E58, E22	6	3
D22	I am concerned that my personal identification information can be easily compromised or stolen	4 articles: E174, E208, E294, E287	6	4
D23	I think most of the latest sports information and related data available on social media are true and reliable	3 articles: E148, E96, E110	52	9
D24	I feel that the smart push sports information can meet the needs of pre-service physical education teachers for learning different sports specialties	2 articles: E48, E4	3	3
D25	I believe that the vehicles of picture, sound, and text are very suitable for the comprehensive development of the professional perceptions of pre-service physical education teachers	3 articles: E77, E233, E166	47	8
D26	I feel that social media allows pre-service physical education teachers to communicate across school and classroom boundaries	16 articles: E148, E96, E110, E223, E206, E56, E121, E182, E43, E264, E300, E194, E209, E157, E283. E195	7	4
D27	I believe that there are resources in social media that meet the needs of pre-service physical education teachers at all levels	4 articles: E46, E75, E268, E282	24	7
D28	I feel that the language translation function has helped pre-service physical education teachers to connect internationally	4 articles: E38, E273, E146	28	4
D29	I think it’s time-consuming to find sports resources that fit in the confusing categorization of resources in social media	3 articles: E9, E19, E225	21	2
D30	I feel that social media provides a database of physical education information that pre-service physical education teachers need	4 articles: E15, E301, E59, E280	12	3
D31	I believe that the wide range of physical education resources in social media has enabled pre-service physical education teachers to make strong connections between their daily lives and physical education	3 articles: E147, E155, E8	5	1
D32	I feel that smart push content can make me inspired about physical education (e.g., health education view, etc.)	3 articles: E242, E16, E36	3	3
D33	I have found that social media allows me to easily collaborate and communicate with in-service physical education teachers	4 articles: E55, E207, E209, E273	3	3
D34	I feel that the atmosphere in the virtual community, topic discussion and other subdivisions about exercise skills is lively	3 articles: E83, E92, E195	7	2
D35	I feel that the search and collection of sports information in social media is easy to organize	1 Article: E34, E49, E289	4	3
D36	I feel that paid sports resources in social media are affordable	1 Article: E70	7	2
D37	I feel that sports videos and graphics are very suitable for pre-service level physical education teachers to build sports representations	2 articles: E166, E200	38	10
D38	I feel it is difficult to receive timely responses from high level physical education teachers or research experts on social media	2 articles: E158, E60, E20	12	8
D39	I feel that social media allows pre-service physical education teachers to easily collaborate on interdisciplinary communication	6 articles: E167, E14, E182, E203, E55, E261	9	2
D40	I was worried that my fellow majors and faculty on campus would misunderstand or make fun of what I was posting on social media	1 Article: E208	43	7
D41	I find physical education resources through social media helpful in reinforcing what I have learned in class	3 articles: E155, E97, E137	29	8
D42	I feel that most of the technical aspects of physical education related sharing in social media are not clearly presented	18 Articles: E95, E231	60	7
D43	I think social media can help break the uneven distribution of resources for physical education	6 articles: E194, E227, E2, E172, E30, E111	5	2
D44	I found that there are many users on social media who are interested in sports and fitness	5 articles: E6, E169, E175, E266, E305	19	4
D45	I feel that the different scales of interaction meet the different communication needs of pre-service physical education teachers	5 articles: E74, E133, E149, E122, E170	1	1
D46	I feel that the effects of learning on social media should avoid the effects of offline noise or other distractions	2 articles: E293, E18	6	2
D47	I think social media lacks a screening mechanism for outdated, poor-quality, fraudulent sports information	3 articles: E234, E9, E235	44	4
D48	I feel like there are a lot of authoritative physical education experts in social media	5 articles: E157, E185, E36, E50, E300	3	3
D49	I feel that most sports knowledge skills on social media are not very credible	3 articles: E235, E156, E296	2	1
D50	I feel that funny emojis on social media meet the social and entertainment needs of pre-service physical education teachers	1 Article: E162	4	2
D51	Can recognize that resources in social media can serve pre-service physical education teachers’ understanding of the details of skill movements	5 articles: E166, E200, E7, E166, E129	20	4
D52	Ability to have the self-discipline needed to concentrate on learning about sports in social media	1 Article: E18	3	3
D53	I feel that positive physical activity messages can ignite my passion for extracurricular exercise	2 articles: E73, E257	3	1
D54	I feel that most of the active sports users are motivated by the intention of selling skills courses or nutritional supplements, etc.	1 Article: E235	4	2
D55	I think the vivid and colorful interface matches the aesthetics of all pre-service physical education teachers	1 Article: E132	4	3
D56	I feel that social media has integrated the information resources needed for my field of study	6 articles: E179, E42, E224, E226, E172, E238	6	3
D57	I think social media features can support multiple perspectives on the endless appeal of sports	2 articles: E166, E82	5	3
D58	I think social media can provide me with scientific and effective exercise training programs	4 articles: E75, E240, E49, E212	3	2
D59	I think pre-service physical education teachers have access to a lot of information content outside of the classroom	3 articles: E30, E48, E239	22	5
D60	I found conflicting explanations of motor skill training methods in different sports social media	3 articles: E156, E29, E296	1	1
D61	I am concerned that asking for unsolicited professional information related to physical education will be considered an invasion of privacy	1 Article: E300	7	3
D62	I feel that other users on social media are friendly toward pre-service physical education teachers	3 articles: E209, E114, E229	12	3
D63	The information on social media about non-sports professionals makes me feel a big cultural difference	3 articles: E275, E269, E281	6	5
D64	I think the number of online users of physical education teachers and related practitioners is huge	4 articles: E157, E170, E31, E188	21	3
D65	I think the knowledge and skills acquired are only applicable to non-physical education majors who are physical education enthusiasts	5 articles: E3, E33, E22, E225, E277	46	7
D66	I think the exchange of physical education-type topics conducted by pre-service physical education teachers is sincere and in-depth	5 articles: E173, E182, E195, E203, E55	23	4
D66	I think the exchange of physical education-type topics conducted by pre-service physical education teachers is sincere and in-depth	5 articles: E173, E182, E195, E203, E55	23	4
D67	Seeing students follow, collect, and forward the important and difficult knowledge of physical education majors makes me feel very stressful	1 Article: E92	21	5
D68	I think social media has solved the paradox of learning and training that many pre-service physical education teachers face	2 articles: E178, E228	6	2
D69	I feel that the sharing of novel exercise skills in social media is more engaging than offline classes	3 articles: E239, E104, E197	27	6
D70	I feel that there are too many categories of information, making it difficult to focus on the content related to my major	2 articles: E225, E154	3	2
	Total	307 articles	1122	22

**Table A3:** Results of categorization of 70 concepts.

Subsidiary Categories	Concepts
	D1 I find graphic videos related to sports or teaching practices in social media smooth, clear and easy to understand
C1	D13 I feel that the typographic interface of social media is clean and clear for pre-service physical education teachers
Interface Perception (4)	D55 I think the vivid and colorful interface matches the aesthetics of all pre-service physical education teachers
	D25 I believe that the vehicles of picture, sound, and text are very suitable for the overall development of pre-service physical education teachers’ professional perceptions
	D20 I find it very easy to operate the video clips and other applications needed to share motor skills
C2	D36 I feel that paid sports resources in social media are affordable
Elemental Perception (4)	D17 Live or recorded video with limited shooting angles may affect the learning effect of technical movements
	D37 I feel that sports videos, graphics, etc. are very suitable for pre-service level physical education teachers to build sports representations
	D7 I think physical education majors and teachers are using social media
	D68 I think social media has solved exactly the paradox of learning and training that many pre-service physical education teachers face
C3	D44 I find that there are many users on social media who are interested in exercise and fitness
Current state perception (6)	D64 I feel that the number of online users of physical education teachers and related practitioners is large
	D48 I feel that there are many authoritative physical education experts in social media
	D2 I think there is a lack of communication boards for issues related to physical education
	D11 I think that in the future, all social media will be interconnected to make physical education resources more accessible
	D31 I believe that physical education resources in social media make a strong connection between the daily lives of pre-service physical education teachers and physical education
C4	D15 I feel that social media will encourage the sharing of knowledge on sports nutrition, sports and fitness skills in the future
Development of perception (6)	D18 I think the future of online sports course resources in social media will be more systematic and complete
	D43 I think social media can help break the unequal distribution of resources for physical education
	D19 I feel that in the future, knowledge about sports skills practice and sports theory will be more and more clearly classified
	D41 I feel that physical education resources through social media are helpful in reinforcing what I have learned in class
	D14 I feel that social media has freed pre-service physical education teachers from the constraints of uniform offline content and teaching schedule
	D12 I feel that the vast amount of sports resources in social media has made school no longer the only gateway for pre-service physical education teachers to acquire knowledge and skills
	D23 I think most of the latest sports information and related data obtained from social media are true and reliable
C5	D24 I feel that smart push sports information can meet the needs of pre-service physical education teachers for different sport-specific learning
Value of information (10)	D27 I think resources in social media can meet the needs of pre-service physical education teachers at different levels
	D56 I feel that social media integrates the information resources needed for my field of study
	D59 I think pre-service physical education teachers have access to a lot of information content outside the classroom
	D53 I feel that positive physical activity messages can ignite my enthusiasm for extracurricular exercise
	D30 I feel that social media provides a database of physical education information needed by pre-service physical education teachers
	D66 I feel that the exchange of physical education-type topics conducted by pre-service physical education teachers is sincere and in-depth
	D16 I found that pre-service physical education teachers were more active in sports topic discussions in an anonymous setting
	D62 I feel that other users in social media are friendly towards pre-service physical education teachers
	D26 I feel that social media allows pre-service physical education teachers to communicate across school and classroom boundaries
C6	D33 I find that social media allows me to easily collaborate and communicate with in-service physical education teachers
Value of interaction (9)	D34 I feel that there is a lively atmosphere of discussion about motor skills in the virtual community, topic discussion and other divisions
	D58 I think social media interaction can provide me with scientifically effective exercise training programs
	D3 I think pre-service physical education teachers are happy to share or exchange motor knowledge skills in social media
	D5 I think pre-service physical education teachers can demonstrate strong professional ethics in their interactions with users
	D39 I feel that social media allows pre-service physical education teachers to easily collaborate on interdisciplinary communication
	D45 I feel that the different scales of interaction meet the different communication needs of pre-service physical education teachers
	D4 I find it difficult to screen the authenticity of basic sports theory or specific sports data in social media
	D70 I feel that there are too many categories of information, making it difficult to focus on the content related to my major
	D6 I feel that the information about the sports skills practice category and the sports theory knowledge category is fragmented
C7	D29 I think it’s time-consuming to find sports resources that fit in the confusing categorization of resources in social media
Information Risk (8)	D9 I feel that there is a lack of authoritative sources of information, such as frontline physical education teachers, physical education research experts, and official sports institutions
	D49 I feel that misleading sports skills lead to incorrect exercise methods and harm to physical health
	D60 I found conflicting explanations of physical skill training methods in different sports on social media
	D65 I feel that the knowledge and skills acquired are only applicable to non-physical education majors who are physical education enthusiasts
	D22 I am concerned that my personal identification information can be easily compromised or stolen
C8	D61 I am concerned that asking for unsolicited professional information related to physical education will be considered an invasion of privacy
Privacy Risks (4)	D40 I am worried that my fellow majors and teachers on campus will misunderstand or make fun of what I post on social media
	D67 Seeing students following, collecting and forwarding the important and difficult knowledge of physical education majors makes me feel very pressured
	D47 I think social media lacks screening mechanisms for outdated, poor quality, and fraudulent sports information
	D28 I feel that the language translation function has helped pre-service physical education teachers connect internationally
	D21 I think the video replay and variable speed features make the dynamic changes in the techniques and tactics of each event easier to understand
C9	D32 I feel that intelligent pushing enables me to generate inspiration for physical education (e.g., critical view of health education, etc.)
Functional Perception (8)	D50 I feel that funny emojis in social media meet the social and entertainment needs of pre-service physical education teachers
	D35 I feel that the search and collection of sports information in social media is easy to organize
	D10 I feel that the division between motor skills and sport theory in social media is unclear
	D57 I feel that the capabilities of social media can support multiple perspectives on the endless appeal of sports
	D46 I feel that the effects of learning in social media should avoid the effects of offline noise or other distractions
	D42 I feel that most of the technical aspects of physical education-related sharing in social media are not clearly presented
	D8 I feel that pre-service physical education teachers who lack teaching experience are prone to misunderstandings or arguments during social media sharing
C10	D63 Social media messages from non-sports professionals make me feel very culturally different
Psychological Risk (8)	D38 I feel it is difficult to receive timely responses from high-level physical education teachers or research experts on social media
	D52 Need to have the self-discipline needed to concentrate on learning about sports in social media
	D54 I feel that most of the active sports users are motivated by the intention of selling skills courses or nutritional supplements, etc.
	D69 I feel that the sharing of novel exercise skills on social media is more engaging than in offline classes but it is easy to get distracted
